# Frequency and leg stiffness adaptation in human vertical hopping before, during and after added load

**DOI:** 10.1242/jeb.250848

**Published:** 2025-12-11

**Authors:** Luke N. Jessup, Moira A. Williams, Alex J. Duman, Juwon Lee, Raksha S. Konanur, Anne K. Silverman, James M. Finley, Monica A. Daley, Alexandra S. Voloshina

**Affiliations:** ^1^ Department of Ecology & Evolutionary Biology, University of California, Irvine, Irvine, CA 92697, USA; ^2^ Division of Biokinesiology and Physical Therapy, University of Southern California, Los Angeles, CA 90033, USA; ^3^ Department of Mechanical and Aerospace Engineering, University of California, Irvine, Irvine, CA 92697, USA; ^4^ Department of Mechanical Engineering, Colorado School of Mines, Golden, CO 80401, USA; ^5^ Quantitative Biosciences and Engineering Program, Colorado School of Mines, Golden, CO 80401, USA; ^6^ Department of Biomedical Engineering, University of California, Irvine, Irvine, CA 92617, USA

**Keywords:** Biomechanics, Bouncing, Mass, Optimisation, Energy, Mechanical demands

## Abstract

Terrestrial animal gaits often use spring-like mechanics to enhance movement economy through elastic energy cycling. Hopping is a relatively simple, constrained task, yet retains essential features of bouncing gaits, requiring cyclic regulation of limb stiffness and generation of high muscle forces to support body weight and enable elastic energy cycling. We investigated how humans adjust hopping frequency and leg stiffness before, during and after experiencing added load. Eighteen participants hopped bipedally for 90 s per trial, with hop frequency and height unconstrained, while kinematic, ground reaction force and ankle muscle electromyographic (EMG) data were collected. We analysed mechanics across four conditions: initial body weight (BWi), two added mass trials (+10% and +20% BW) and final body weight (BWf). With added mass, participants increased leg stiffness and maintained a consistent hopping frequency (∼2.15 Hz); yet, when returning to BWf, the elevated leg stiffness was maintained and hopping frequency increased (to ∼2.36 Hz) and reduced centre of mass (CoM) work per hop. BWf adaptations were driven by greater ankle stiffness, leading to less ankle work. Adaptation rates were consistent across trials, with steady-state mechanics reached in ∼30–40 s. Muscle coactivation decreased following BWi. Triceps surae mean EMG was unchanged with added mass and reduced in BWf. Similar patterns of adaptation were observed in bouncing without an aerial phase. Substantial inter-individual variability was observed in preferred hopping mechanics and adaptation strategy. Together, added mass and increased task familiarity led participants to recalibrate their hopping strategy. Based on literature evidence, the adaptations may align with reduced metabolic cost.

## Teaser

**Summary:** Following exposure to added mass, humans adapt their preferred hop mechanics, increasing hop frequency and leg stiffness – adjustments that, based on literature evidence, may result in reduced metabolic cost.

Economic movement often involves rhythmic, cyclical motions that enable passive cycling of mechanical energy to minimise active muscle work (Cavagna, 1975; Cavagna et al., 1977; Heglund et al., 1982). Walking, running and hopping all exhibit characteristic ‘bouncing’ motion of the body centre of mass (CoM) that can be modelled as a point-mass bouncing on a simple spring (Alexander and Vernon, 1975; McMahon and Cheng, 1990; Blickhan, 1989; Farley et al., 1991, 1993; Alexander, 2002; Geyer et al., 2006). Among these gaits, vertical hopping provides an elegantly simplified model for studying bouncing locomotion as motion occurs only in the vertical direction, with no forward velocity (Farley et al., 1991, 1993; Moritz and Farley, 2004). This simplifies the dynamics, and facilitates integrative study of muscle, joint and whole-body mechanics. Yet, despite this apparent simplicity, successful hopping requires precise coordination to meet multiple demands: controlling limb stiffness to regulate frequency; generating sufficient vertical forces to support body weight; maintaining balance and position in space; and modulating mechanical energy to sustain steady hopping.

The spring-like behaviour of the limb during hopping emerges from the integration of muscle activation and elastic energy storage and return in tendons. Elastic mechanisms in muscle–tendon systems play an essential role in enabling economic bouncing motions. Musculotendinous compliance cycles elastic energy and regulates the forces and energy flow between the environment, the body and the muscles (Alexander and Bennet-Clark, 1977; Alexander, 2002; Roberts and Azizi, 2011). In human bouncing motions, the ankle joint acts as the primary contributor to regulating leg stiffness for spring-like whole-limb function (Farley and Morgenroth, 1999), with the triceps surae muscles contracting nearly isometrically during early stance while the Achilles tendon stores and returns elastic energy (Hof et al., 2002; Lichtwark and Wilson, 2005, 2007; Monte et al., 2021; Swinnen et al., 2022). When humans self-select their hopping frequency, individual preferences can range from 2.0 to 2.9 Hz (Jones and Watt, 1971; Taylor, 1985; Farley et al., 1991; Granata et al., 2002; Jessup et al., 2023; Allen et al., 2022), which may reflect a balance between competing demands including metabolic cost, muscle fatigue and perceived effort.

Regulation of leg stiffness is fundamental to hopping dynamics and influences potential trade-offs among potentially competing neuromechanical priorities. Hopping frequency depends on both aerial phase duration and stance phase duration (Morin et al., 2007). The aerial phase duration is determined by vertical take-off velocity, assuming ballistic motion of the body CoM. Stance duration depends on the resonant frequency as approximated as a linear mass–spring system. The frequency (*f*) of the resonant bounce in the stance phase is determined by mass (*m*) and vertical stiffness (*k*), according to the equation:
(1)

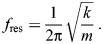
Body mass (*m*) is usually considered constant, whereas vertical stiffness (*k*) varies depending on muscular effort and can be adjusted from hop to hop to control stance duration. Humans vary vertical stiffness in response to changes in substrate properties, to maintain a consistent combined stiffness of the leg and substrate (Ferris et al., 1998; Moritz and Farley, 2004). When humans are asked to hop at a range of fixed frequencies above and below their preferred frequency, they maintain a linear, spring-like function of the limb at higher-than-preferred frequencies but lose this spring-likeness at lower-than-preferred frequencies (Farley et al., 1991). At low frequencies, leg stiffness becomes non-linear, such that the minimum in CoM height does not align in time with the peak force as would be expected for a simple spring–mass system (Farley et al., 1991). This non-linearity is accompanied by muscle fascicles actively absorbing and performing more work relative to the amount of work recycled by tendon (Jessup et al., 2023; Monte et al., 2021). Thus, preferred hop frequencies, and therefore leg stiffness, are thought to optimise elastic energy cycling.

Previous studies examining brief single-legged and double-legged hopping trials lasting 5–10 s have found that added body mass (+5%, +10%, +20%) does not affect leg stiffness (Carretero-Navarro and Márquez, 2016; Donoghue and Steele, 2009), which corresponds with a decrease in hop frequency (Austin et al., 2002). However, these short trials may not have allowed sufficient time for optimisation of hopping strategy under novel loading conditions. During running trials lasting 2 min, increasing body mass (+10%, +20%, +30%) led to increased leg stiffness, with no change in stride frequency, resulting in higher peak vertical forces (Silder et al., 2015). Although the cost of running increases with added mass (Epstein et al., 1987; Marsh et al., 2006; Teunissen et al., 2007), performing 60 s of running with added body mass (+20%) during warm-up elicited greater leg stiffness and improved running economy during a subsequent 5 min submaximal run at normal body mass (Barnes et al., 2015); stride frequency and peak forces were not reported. The effects of added mass on gait also vary across organisms: for example, horses incur metabolic costs that scale with load and lower their preferred trot-to-gallop transition speed to accommodate earlier critical musculoskeletal loading (Farley and Taylor, 1991), whereas wallabies show no change in cost or hop frequency when carrying young, owing to the energy storage capacity of their hindlimb tendons (Baudinette and Biewener, 1998). Collectively, these findings suggest that added mass may alter preferred mechanics both during its application and after the load is removed, potentially serving as a simple yet powerful intervention to probe motor adaptation.

Here, we investigated how humans regulate leg stiffness, ground reaction force, mechanical work and muscle excitation during 90 s double-legged vertical hopping trials with added mass equivalent to 10% and 20% of body weight. This time scale was selected to allow observation of adaptation while avoiding excessive fatigue. We tested the hypothesis that humans increase leg stiffness proportional to added mass to maintain consistent hopping frequency. This adaptation would require greater peak forces but might reduce total mechanical work and muscle activation per unit of time. We also tested the hypothesis that humans would return to their original preferred frequency in a final unloaded trial following exposure to both added mass conditions. A return to the initial preferred frequency would suggest that self-selected leg stiffness and hopping frequency have been optimised for consistent neuromechanical criteria, such as energy minimisation or fatigue avoidance.

## MATERIALS AND METHODS

### Participants

Eighteen healthy adults (10 male, 8 female), free of musculoskeletal injury or neuromuscular disorders, age 31±11 years, mass 65±11 kg, leg length 88±6 cm and height 172±112 cm (means±s.d.), gave their written informed consent to participate in this study. The study protocol was approved by and conducted in accordance with the University of California, Irvine Institutional Review Board (#20195298), and conformed to the standards set by the Declaration of Helsinki.

### Experimental protocol

Participants were instructed to hop in place on both feet, with the left and right foot landing on separate force plates (2000 Hz; 9260AA, Kistler, Winterthur, Switzerland). Participants hopped for 90 s under the instruction to hop at a comfortable, continuous rhythm and pace that they could sustain for the entire trial, keeping their arms rigidly at their sides. Hop frequency and hop height were unconstrained. Hop width (i.e. foot position) was also unconstrained except for the requirement to maintain each leg on a separate force plate. Trials were performed in the same sequence for all participants and included: (1) an initial body weight (BWi) trial; (2) 10% added body mass (BW10); (3) 20% added body mass (BW20); and a final body weight (BWf) trial. Participants rested for at least 3 min between trials, standing but free to stretch or shake their legs. We excluded the data from 1 of the 18 participants because they were unable to sustain a cyclical hopping rhythm for the full length of each trial.

On a separate day following the hopping protocol, each participant performed the same sequence of conditions, in the same order, while bouncing in place on both feet (i.e. without an aerial phase). Participants were instructed to oscillate vertically at a comfortable, sustainable rhythm and pace, with bounce frequency and height left unconstrained. We excluded bouncing data from 5 of the 18 participants because of an inability to sustain a cyclical bouncing rhythm for the full length of each trial. Data collection and analysis procedures for bouncing were equivalent to those described below for hopping. The full bouncing methods and results are provided in the Supplementary Materials and Methods, as these data serve a complementary role to the primary hopping analyses.

### Kinematics and kinetics

Participants were instrumented with a modified Vicon Nexus Lower Body Plug-In-Gait marker set, with reflective markers placed on the left and right 1st, 2nd and 5th metatarsophalangeal joints, lateral and medial malleolus, posterior calcaneus, lateral mid-shank, lateral joint rotation centre of the knee, lateral mid-thigh, anterior superior iliac spine (ASIS) and posterior superior iliac spine (PSIS). Eight motion capture cameras (Vero, Vicon, Oxford, UK) were used to capture the position of the markers at 100 Hz, time-synchronised with the ground reaction force (GRF) data in Nexus motion analysis software (Vicon). Raw marker trajectories and GRF data were then filtered using a zero-lag fourth-order low-pass Butterworth filter with 25 Hz cut-off frequency. These filtered data were then processed via the Plug-In-Gait Dynamic Pipeline within Nexus to calculate joint angles, moments and powers. A custom MATLAB (MathWorks, Natick, USA) script was then used to export kinematic and kinetic data for analysis.

All CoM- and joint-level parameters were measured for each hop in each trial. Landing and toe-off events were indexed from GRF data using a 30 N threshold, allowing us to compute hop frequency, stance time, aerial time and duty factor. Resonant frequency was calculated as:
(2)

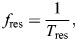
where *T*_res_ is the resonant period, defined as twice the duration of stance during which the vertical GRF exceeded one body weight (Farley et al., 1991). Average leg stiffness (*k*_leg_) during stance was calculated as the ratio of peak vertical GRF (*F*_peak_) and the vertical displacement of the centroid of the pelvis markers (i.e. the average vertical displacement across the ASIS and PSIS markers; an estimate of the CoM). CoM position was estimated from the pelvis markers to avoid drift associated with double-integration of force plate data (Cavagna, 1975) during the 90 s trials. Vertical CoM displacement was measured from the point when vertical GRF equalled one body weight through until *F*_peak_ and was used as a proxy for leg compression (Δ*L*):
(3)

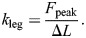
CoM power over the entire hop cycle (from touch-down to the end of the subsequent aerial phase) was calculated as the product of vertical GRF and the vertical velocity of the CoM. CoM work per hop was calculated by integrating CoM power over time. Notably, vertical CoM displacement may have been overestimated having been derived from pelvis marker positions (Mudie et al., 2017), potentially leading to systematic overestimations of CoM work and underestimations of *k*_leg_.

For the ankle, knee and hip, average torsional joint stiffness (*k*_joint_) during the first half of the stance phase was calculated as the ratio of the change in joint moment (Δ*M*_joint_) and joint angular displacement in the sagittal plane (Δθ_joint_) from touch-down to the instant that the joint was maximally flexed:
(4)

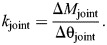
Joint power was calculated as the product of joint moment and angular velocity in the sagittal plane, and joint work over the entire hop cycle was calculated by integrating joint power over time.

### Muscle excitation

Surface electromyography (EMG) signals were recorded from lateral gastrocnemius (LG), medial gastrocnemius (MG), soleus (SOL) and tibialis anterior (TA). Recording sites were prepared by shaving and then cleaning the skin with alcohol wipes. Pre-amplified single differential electrodes (Trigno Avanti, Delsys, Boston, MA, USA) were placed over each muscle, according to SENIAM guidelines (Hermens et al., 2000), and secured to the leg using self-adherent bandage (Coban, 3M, St Paul, MN, USA). EMG signals were hardware filtered with a bandwidth of 20–450 Hz and recorded in Nexus at 2000 Hz. The signals were processed in MATLAB where the DC offset was removed using a fourth-order high-pass Butterworth filter at 25 Hz, followed by signal rectification, and finally a fourth-order low-pass Butterworth filter at 10 Hz to create an envelope. We calculated the mean EMG of the envelope for each hop cycle, which approximates the average recruitment of musculature over time. For each participant, mean EMG values for each muscle were normalized to the mean EMG value of that muscle throughout their BWi condition.

### Statistics

All statistical analyses were performed on dimensionless quantities. SI quantities were converted to dimensionless quantities using body mass and leg length (Tables S1 and S2) and gravitational acceleration as per the conventions introduced by McMahon and Cheng (1990) [see table 2 in Daley and Birn-Jeffery (2018) for a summary of the relevant scaling factors]. Table values are presented in dimensionless units (Tables S4 and S6 contains the equivalent SI units based on the average participant body mass and leg length). Figures are presented in dimensionless units and in SI units based on average participant characteristics.

We were interested in the time required for participants to adapt to each experimental condition. One of the main task-level priorities of the nervous system during vertical hopping is the stabilisation of the magnitude of vertical force production (Selgrade and Chang, 2015; Yen et al., 2009; Yen and Chang, 2010). Thus, changes in peak vertical force during hopping provide an indication of adaptation to different experimental conditions. To approximate the time taken for peak vertical force to stabilise, we used a method similar to McAllister et al. (2021) for step frequency adaptation during walking. We first applied a 15 s moving average to identify the period of peak vertical force data with the lowest standard deviation, representing the ‘steadiest’ portion of the trial. We then identified the first time point of the entire trial when the peak vertical force fell within ±1 s.d. of the mean of this steady period for at least 2.5 s (equivalent to at least 4 consecutive hops). This time point defined the ‘settling time’. To assess differences in adaptation rate between conditions, we performed a one-way ANOVA with Bonferroni correction.

**Fig. 1.**
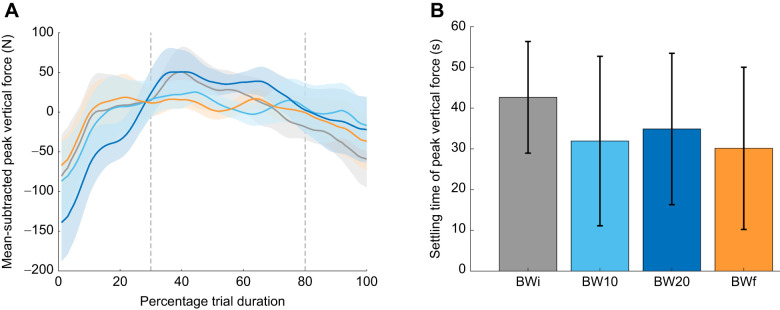
**Adaptation of peak vertical force across hopping conditions.** Participants hopped under conditions of: initial body weight (BWi; grey), body weight +10% (BW10; light blue), body weight +20% (BW20; dark blue) and final body weight (BWf; orange). (A) Mean-subtracted peak vertical force data, time normalised as a percentage of trial duration. Solid lines show the group mean, with shaded regions representing ±95% confidence intervals (CI). Vertical dashed lines at 30% and 80% of trial duration indicate the ‘steady’ period used for statistical analyses. (B) Settling time of peak vertical force adaptation, shown as the group mean±s.d.

We observed that parameters tended to vary during the first 30% and last 20% of each trial, keeping relatively steady between 30% and 80% of the trial (Fig. 1A). Numerous studies have successfully measured steady-state metabolic energy expenditure during double-legged vertical hopping by having participants hop for 4–5 min per trial (Allen et al., 2022; Farris and Sawicki, 2012; Grabowski and Herr, 2009; Gutmann and Bertram, 2013; Jessup et al., 2023; Monte et al., 2021). Although these studies were conducted without added mass, aside from bilateral ankle exoskeletons in some cases (mass unreported), unsteadiness during the final 20% of each trial may indicate that our participants had lower fitness levels and/or experienced greater fatigue, that participants reacted to updates given on the remaining trial duration, or that further adaptation would have occurred over a longer trial duration. Nevertheless, all subsequent analyses focused on the ‘steady-state’ period between 30% and 80% of the trial. Analyses were conducted on values averaged within each 10% interval of the steady-state period (i.e. 30–80% of the trial, divided into ten equal intervals representing 10–100% of the steady-state period), with analyses performed across these averaged values rather than treating each individual interval independently. We used linear mixed-effects (LME) ANOVA through the MATLAB Statistics Toolbox, with trial condition and sex as categorical fixed effects, age as a continuous covariate factor, and individual as a random effect. Model 1 included only the intercept and individual as a random effect [Y∼1+(1|ind)], serving as a reference model and null hypothesis among the multiple alternative models that we compared:
Model 1

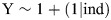

Model 2

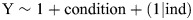

Model 3

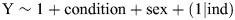

Model 4

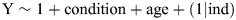

Model 5

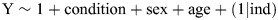

Model 6

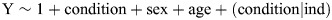


Candidate models were compared based on AIC, BIC and adjusted *R*^2^ tests, which supported the selection of Model 6 as the most comprehensive model. For clarity, Model 6 accounts for both between-individual differences in baseline values (random intercepts) and variability in how individuals respond to each condition (random slopes). For each variable analysed, we calculated the ANOVA test statistics. After false discovery rate (FDR) correction (*P*≤0.0209 to maintain FDR of 5%), we determined whether there was a significant effect of condition or sex. Although age was included as a potential confounding factor because it influenced model selection criteria, we do not report age effects because of the highly non-Gaussian age distribution in our sample (Table S1), and including age does not substantially alter the timing or interpretation of the fixed effects. We also calculated the pairwise difference (mean±95% confidence interval, CI) between the BWi and BW10, BW20 and BWf conditions using *post hoc* comparisons with Bonferroni correction (*P*≤0.05), and computed Cohen's *d* to quantify effect sizes for these pairwise differences. Because Model 6 included individual random effect coefficients for all conditions, we examined the correlations among random effect coefficients to explore the variation in how individuals responded to the conditions.

## RESULTS

A fast period of peak vertical force adaptation occurred during the first ∼10% of each 90 s trial (Fig. 1A). This was followed by a slower phase of adaptation lasting an additional ∼20–30%, before data plateaued (Fig. 1A,B). Throughout the remainder of each trial, a slow decline in peak vertical force was observed. Although not significant (*P*=0.216), the rate of adaptation tended to be slower in the BWi condition.

### CoM mechanics

**Fig. 2.**
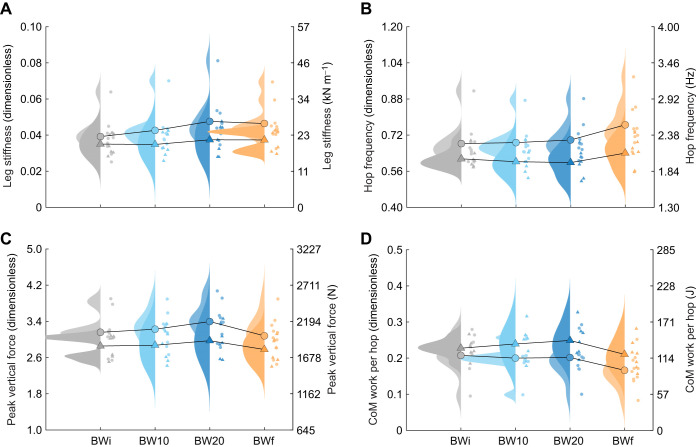
**Centre of mass (CoM) mechanics across hopping conditions.** Raincloud plots (combining a probability density and scatter of individual data points; Allen et al., 2021) of male (circles; lighter rainclouds) and female (triangles; darker rainclouds) (A) leg stiffness, (B) hop frequency, (C) peak vertical force and (D) CoM work per hop in response to the initial body weight (BWi; grey), body weight +10% (BW10; light blue), body weight +20% (BW20; dark blue) and final body weight (BWf; orange) hopping condition. Individual points are the mean for each participant, and the mean values across participants are connected with lines. Data are presented in dimensionless quantities, with the secondary *y*-axes corresponding to SI unit equivalents based on the average participant mass and leg length.

Participants increased leg stiffness in response to added mass, and then following removal of the added mass, leg stiffness was greater in the BWf condition than in the BWi condition (*P*<0.001; Table 1, Fig. 2A). This behaviour resulted in a similar hop frequency between BWi, BW10 and BW20 (2.15–2.17 Hz), but a greater hop frequency in BWf (2.36 Hz; *P*<0.001; Fig. 2B). Stance time increased and aerial time decreased with added mass (*P*<0.001), while both stance and aerial times decreased in BWf (*P*<0.001). Resonant frequency was lower in BW10 and BW20 than in BWi, but greater in BWf (*P*<0.001; Table 1). Peak vertical force only differed from BWi in BW20 (*P*<0.001; Fig. 2C), and thus, the greater leg stiffness in BWf (23 kN m^−1^) relative to that in BWi (20 kN m^−1^) was driven by smaller CoM displacement. CoM work per hop increased with added mass, but was 14% smaller in BWf than in BWi (*P*<0.001; Fig. 2D).

**Table 1.** Linear mixed-effects ANOVA results on hopping mechanics

ANOVA *F*-stat		Variable (dimensionless)	Condition: pairwise difference from BWi [mean±95% CI (effect size)]		
Condition	Sex		BW10	BW20	BWf
**14.70**	**7.11**	Hop frequency	−0.0041±0.0066 (−0.09)	0.0020±0.0066 (0.07)	**0.0582±0.0066 (1.08)**
**43.82**	**24.75**	Stance time	**0.0340±0.0073 (0.81)**	**0.0478±0.0073 (0.66)**	−**0.0397±0.0073 (**−**0.74)**
**8.81**	1.90	Aerial time	−**0.0237±0.0016 (**−**0.54)**	−**0.0461±0.0016 (**−**0.99)**	−**0.0767±0.0016 (**−**1.05)**
**44.72**	**24.63**	Resonant frequency	−**0.0422±0.0076 (**−**0.93)**	−**0.0530±0.0076 (**−**0.77)**	**0.0606±0.0076 (0.79)**
**14.39**	4.75	Peak vertical force	0.0513±0.0718 (0.23)	**0.1834±0.0718 (0.61)**	−0.0699±0.0718 (−0.25)
**7.32**	0.55	Leg stiffness	**0.0018±0.0005 (0.53)**	**0.0060±0.0005 (1.02)**	**0.0051±0.0005 (0.85)**
**23.73**	2.14	CoM work per hop	0.0027±0.0036 (0.01)	**0.0065±0.0036 (0.19)**	−**0.0299±0.0036 (**−**0.95)**
2.61	**8.92**	Ankle stiffness	0.0113±0.0146 (0.19)	**0.0400±0.0146 (0.49)**	**0.0555±0.0146 (0.52)**
0.92	2.47	Ankle peak moment	0.0038±0.0098 (0.02)	**0.0106±0.0098 (0.23)**	0.0029±0.0098 (−0.01)
**7.64**	5.77	Ankle positive work	−0.0015±0.0034 (−0.23)	−0.0011±0.0034 (−0.14)	−**0.0084±0.0034 (**−**0.79)**
**6.54**	4.88	Ankle negative work	−0.0029±0.0034 (−0.26)	−**0.0037±0.0034 (**−**0.33)**	**0.0049±0.0034 (0.64)**
**10.94**	3.20	LG mean EMG	−0.0484±0.0536 (−0.20)	−**0.1197±0.0536 (**−**0.39)**	−**0.2370±0.0536 (**−**1.02)**
**6.77**	0.16	MG mean EMG	−0.0359±0.0447 (−0.23)	0.0112±0.0447 (−0.17)	−**0.0683±0.0447 (**−**0.47)**
**11.68**	2.72	SOL mean EMG	0.0354±0.0503 (0.39)	0.0440±0.0503 (0.33)	−**0.1298±0.0503 (**−**0.89)**
**13.94**	1.99	TA mean EMG	−**0.2494±0.0724 (**−**1.16)**	−**0.2187±0.0724 (**−**0.90)**	−**0.3000±0.0724 (**−**1.44)**

Condition, age and sex were fixed effects, with (condition|individual) as random effects (Model 6, see Materials and Methods). Degrees of freedom: condition d.f.=3, age d.f.=1, sex d.f.=1, error d.f.=403. Bold indicates statistical significance. BWi, initial body weight; BW10, body weight +10%; BW20, body weight +20%; BWf, final body weight; CoM, centre of mass; LG, lateral gastrocnemius; MG, medial gastrocnemius; SOL, soleus; TA, tibialis anterior; EMG, electromyography.

### Joint mechanics

**Fig. 3.**
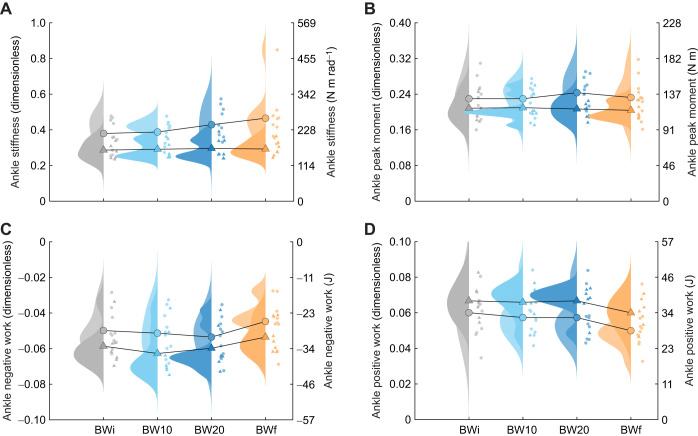
**Ankle joint mechanics across hopping conditions.** Raincloud plots of male (circles; lighter rainclouds) and female (triangles; darker rainclouds) (A) ankle stiffness, (B) ankle peak moment, (C) ankle negative work and (D) ankle positive work in response to the BWi (grey), BW10 (light blue), BW20 (dark blue) and BWf (orange) hopping condition. Individual points are the mean for each participant, and the mean values across participants are connected with lines. Data are presented in dimensionless quantities, with the secondary *y*-axes corresponding to SI unit equivalents based on the average participant mass and leg length.

**Fig. 4.**
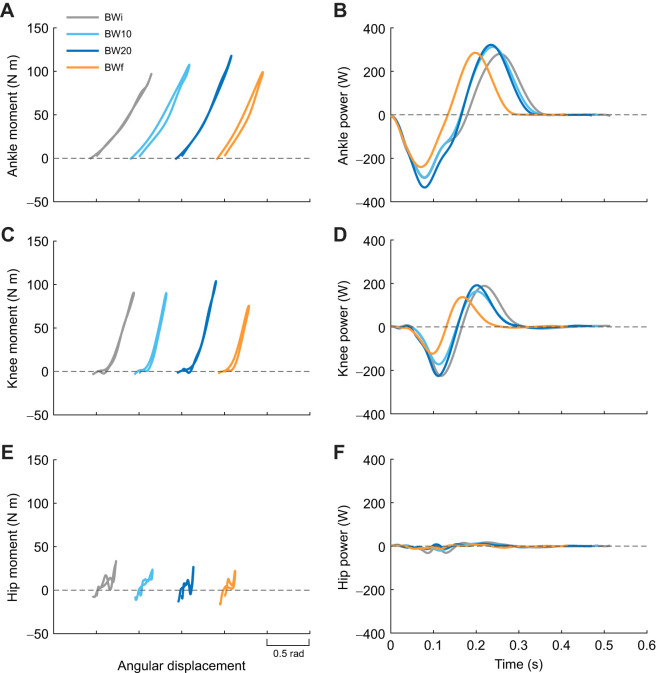
**Joint moment and power across hopping conditions.** Joint moment versus angular displacement for (A) ankle, (C) knee and (E) hip during stance in response to the BWi (grey), BW10 (light blue), BW20 (dark blue) and BWf (orange) hopping condition for a representative participant (sex=male, age=21 years, mass=63.2 kg, leg length=0.93 m). Note, the relative horizontal positions of the curves is not meaningful, and for all joints, moving right on the *x*-axis indicates flexion. Joint power across the entire hop cycle is also presented for (B) ankle, (D) knee and (F) hip.

Despite not having a significant effect of condition (*P*=0.051; Table 1), the ankle was the only joint to have significant pairwise differences in stiffness from BWi, with greater ankle stiffness in BW20 and BWf (*P*<0.001; Fig. 3A). As for CoM dynamics, peak ankle moment only differed from BWi in BW20 (*P*<0.001; Fig. 3B), and so the greater ankle stiffness in BWf (219 N m rad^−1^) versus BWi (186 N m rad^−1^) was driven by smaller ankle angular displacement (*P*<0.001; Table S1). There were differences in negative work at the ankle (*P*=0.027), knee (*P*=0.011) and hip (*P*<0.001) in BW20 compared with BWi. However, the differences in joint work were larger between BWi and BWf, with reductions in both the negative and positive work of each joint (*P*<0.001; Table S3; Fig. 3C,D; Fig. 4B,D,F).

### Muscle excitation

**Fig. 5.**
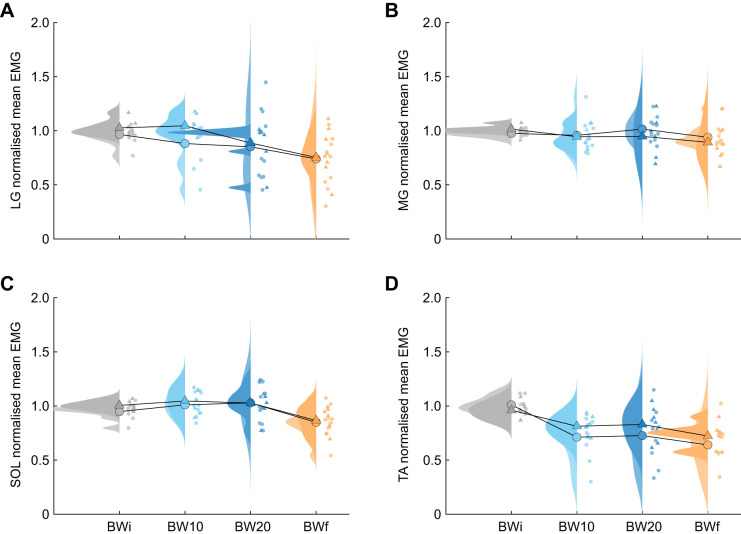
**Ankle muscle excitation across hopping conditions.** Raincloud plots of male (circles; lighter rainclouds) and female (triangles; darker rainclouds) mean electromyography (EMG) from (A) lateral gastrocnemius (LG), (B) medial gastrocnemius (MG), (C) soleus (SOL) and (D) tibialis anterior (TA) in response to the BWi (grey), BW10 (light blue), BW20 (dark blue) and BWf (orange) hopping condition. Individual points are the mean for each participant, and the mean values across participants are connected with lines.

There was a significant effect of condition on the mean EMG of LG, MG, SOL and TA (*P*<0.001; Table 1). Compared with the BWi condition, LG mean EMG decreased in BW20, while TA mean EMG decreased in both BW10 and BW20 (*P*<0.001; Table 1, Fig. 5A,D; Fig. S1A,D). The mean EMG of all muscles was lower in the BWf condition than in the BWi condition (*P*<0.001; Table 1, Fig. 5; Fig. S1).

### Individual variation

The variance explained by individual as a random effect in the LME was substantial, and higher than the variance explained by condition across almost all variables. Individual as a random effect explained 73–84% of the variance in CoM mechanics, whereas condition as an added factor explained a further 7–16%. To explore the structure of individual variation, we examined the correlations among the random effect coefficients between the BWi and BWf conditions for the variables presented in Table 1. The correlations were strong for LG (*R*=0.96), MG (*R*=−0.97) and SOL (*R*=−0.86) mean EMG, moderate for stance time (*R*=−0.53), peak vertical force (*R*=−0.47), CoM work per hop (*R*=−0.31), resonant frequency (*R*=−0.53), ankle peak moment (*R*=−0.44), ankle positive work (*R*=−0.61) and TA mean EMG (*R*=−0.51), and weak for hop frequency (*R*=−0.11), aerial time (*R*=−0.08), leg stiffness (*R*=−0.22), ankle stiffness (*R*=−0.01) and ankle negative work (*R*=−0.09). These results and the spread of data observed in Figs 3, 4 and 5 indicate substantial interindividual variability.

Compared with the female participants, males exhibited a faster hop frequency (*P*=0.007), reduced stance time and higher load rate (*P*<0.001), reduced ankle (*P*<0.001), knee (*P*=0.017) and hip (*P*=0.004) angular displacements, higher ankle stiffness (*P*=0.003), and reduced hip peak power (*P*=0.013) and hip positive (*P*<0.001) and negative work (*P*=0.001). There was no effect of sex on mean EMG.

## DISCUSSION

With added mass, participants increased leg stiffness to maintain a hop frequency similar to BWi. Although hop frequency was preserved, stance time increased while aerial time (and thus hop height) decreased, with a lower predicted resonant frequency of the spring–mass system. After removing the added mass, leg stiffness remained elevated, stance and aerial times decreased, and hop frequency increased, contrary to the hypothesis that participants would return to their original preferred frequency. Compared with BWi, the higher hop frequency in BWf led to lower CoM work per hop. Consistent with other hopping research (Hobara et al., 2011; Farley and Morgenroth, 1999; Farley et al., 1998), within the range of hop frequencies observed (1.7–3.3 Hz), changes in leg stiffness were driven by changes in ankle stiffness. Ankle stiffness increased with added mass exposure and remained elevated afterward, leading to reduced positive and negative ankle work and mean EMG of ankle musculature in BWf compared with BWi. Thus, added mass, together with more experience hopping, allowed participants to converge on a new hopping strategy, and it is possible that this strategy is closer to the energetic optimum (Gutmann and Bertram, 2013; Jessup et al., 2023).

The adaptations observed during the application of added load, and following its removal, are consistent with findings from studies of running with added load. Added mass during running increases leg stiffness (Silder et al., 2015), and this elevated leg stiffness can persist after the load is removed (Barnes et al., 2015). Barnes et al. (2015) argued that post-activation potentiation – a phenomenon in which heavy exercise preconditions the muscle to induce acute performance gains (Blazevich and Babault, 2019) – was primarily responsible for their results. Unfortunately, neither their protocol nor ours spanned a sufficient time frame to determine whether the effects of added mass reflected transient potentiation or a more sustained adaptation. In addition, hopping with and without added mass are novel movement tasks for many individuals, and thus an optimised control strategy may not be fully developed (Croft et al., 2019) – changes in preferred mechanics between BWi and BWf, and the possibility that resonance was not fully exploited under added mass, are perhaps unsurprising. Studies of human locomotion involving exploration-based learning have demonstrated shifts in self-selected behaviour after exposure to sufficiently large stimuli (Finley et al., 2013; Reisman et al., 2005; Sánchez et al., 2019; Selinger et al., 2015, 2019). Both the time taken to reach steady state within each condition (30–40 s; Fig. 1) and the persistence of changes in stiffness after load removal suggest some level of motor learning. The 5–10 s trials used in similar hopping protocols may have been too brief to capture adaptation to added mass, potentially explaining why previous studies reported no change in vertical stiffness (Carretero-Navarro and Márquez, 2016; Donoghue and Steele, 2009) and a decrease in hop frequency (Austin et al., 2002) under added load.

There are a few possible explanations for why we observed a reduction in LG and TA mean EMG, alongside a lack of change in MG and SOL mean EMG, with added mass exposure. The higher degree of muscle co-contraction in the BWi condition may reflect participants' lack of practice with hopping (Fig. 5; Fig. S1). Studies examining individual differences in motor coordination strategies during both lower- and upper-body movement tasks have shown that greater task experience leads to reduced co-contraction (Beveridge et al., 2020; Huang et al., 2012; Miura et al., 2013). Thus, our participants may have developed an internal model of dynamic tuning that enabled them to reduce TA activity and, in turn, lessen the activation demands on the triceps surae. Still, the larger joint moments with added mass suggests that muscles may have operated closer to optimal lengths for force production, or that greater muscle stretch induced by the added mass led to force enhancement (Hahn and Riedel, 2018; Pinniger and Cresswell, 2007). We did not observe adaptation of EMG within the BWi condition, suggesting that the shift in EMG relates to the experience of hopping with added mass (Fig. S2). Added mass may have led to a redistribution of activation demands toward the knee musculature. Unfortunately, we did not collect the fascicle or EMG data required to further evaluate these possibilities. Nevertheless, mean EMG was lower across all measured muscles in the BWf condition compared with the BWi – whether due to added mass, increased task familiarity or both.

**Fig. 6.**
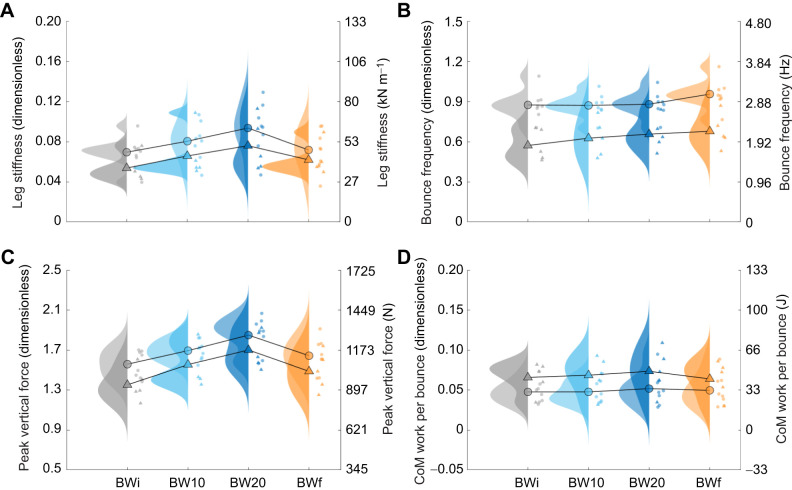
**CoM mechanics across bouncing conditions.** Raincloud plots of male (circles; lighter rainclouds) and female (triangles; darker rainclouds) (A) leg stiffness, (B) bounce frequency, (C) peak vertical force and (D) CoM work per bounce in response to the BWi (grey), BW10 (light blue), BW20 (dark blue) and BWf (orange) bouncing condition. Individual points are the mean for each participant, and the mean values across participants are connected with lines. Data are presented in dimensionless quantities, with the secondary *y*-axes corresponding to SI unit equivalents based on the average participant mass and leg length.

To test whether the findings from hopping persisted in a more constrained task with reduced physical demands, we performed the same experiment during bouncing (no aerial phase; further details provided in the Supplementary Materials and Methods). With added mass, participants increased peak vertical force and leg stiffness, maintained a similar bounce frequency and triceps surae mean EMG, and reduced TA mean EMG (Fig. 6; Table S5, Figs S3 and S4). Then, in the BWf condition, peak vertical force and leg stiffness decreased towards baseline, bounce frequency increased, CoM work per bounce and TA mean EMG showed no change, and triceps surae mean EMG decreased.

Raburn et al. (2011) also used an added mass manipulation during a cyclical bouncing task; however, they found that added mass caused a reduction in preferred frequency, rather than no change. This discrepancy may be a result of their participants performing the task while reclined on a sled, which will have made the task more novel and constrained compared with our experimental setup. Notably, bouncing data were excluded from 5 of our 18 participants due either to an inability to sustain rhythmic bouncing for the duration of each trial or to an unanticipated interpretation of the task instructions resulting in extremely small cyclical oscillations, approximating near-isometric contractions. Nevertheless, the trends were largely consistent with the hopping data, and we were particularly intrigued by the increase in both hop and bounce frequency in the BWf condition compared with the BWi condition.

Humans tend to prefer hop and bounce frequencies that are lower than energetically optimal. For example, on average, humans prefer to hop at ∼2.5 Hz, while the energetic minimum occurs at ∼3.0 Hz (Jessup et al., 2023). Likewise, humans prefer to bounce at ∼2.4 Hz, whereas the energetic minimum lies at ∼2.65 Hz (Merritt et al., 2012). While it is debatable whether energy expenditure is the main criterion driving self-selected movement, it may have contributed to the increase in preferred hop and bounce frequencies observed following added mass exposure. This energy minimisation is supported by the reductions we observed in CoM work during hopping and in mean EMG during both hopping and bouncing. Minimising energy expenditure may be balanced against a preference for distributing fatigue between ankle plantarflexors and knee extensors (McDonald et al., 2022; Miller et al., 2012; Neptune and Hull, 1999) – higher frequencies likely cause relatively more fatigue of the ankle plantarflexors (Farris and Sawicki, 2012). This balance could be achieved through a combination of proprioceptive and nociceptive feedback (Amann et al., 2013; Hayes et al., 2006; Raburn et al., 2011; Windhorst, 2007), helping to identify movement patterns that optimise elastic energy cycling and minimise the accumulation of metabolites within muscle fibres. The lack of an aerial phase in bouncing probably limits the solution space for optimising these variables, contributing to the differences between the adaptation response of hopping and bouncing.

We observed a high degree of interindividual variability in hopping and bouncing dynamics, consistent with previous findings [see fig. 3A from Jessup et al. (2023) and fig. 4 from Merritt et al. (2012)]. We accounted for variation due to body size alone by converting all variables to dimensionless quantities for statistical analysis (McMahon and Cheng, 1990; Daley and Birn-Jeffery, 2018). Additionally, males tended to hop at a higher frequency and with a stiffer posture. However, a range of additional physical and cognitive factors may have contributed to the observed variability, including differences in the relative stiffness or strength of muscle–tendon complexes (Arampatzis et al., 2007; Lichtwark and Wilson, 2008) or in cognitive traits such as novelty seeking, risk propensity or impulsivity (Wang et al., 2015; Kirschmann et al., 2025). Such cognitive factors can lead to individual differences in the tendency to explore solution spaces, leading to motor adaptation in novel conditions, as demonstrated by Selinger et al. (2019), who used exoskeletons to apply resistive torques at the knee to guide individuals toward a new energetically optimal walking gait. Their findings revealed that while some individuals spontaneously optimised in novel conditions, others needed to be encouraged to explore the parameter space by being exposed to varying parameter combinations around the optimum. Age may also have been a source of variation, but our sampling was not systematic enough to allow a formal test of this effect.

The mechanistic interpretation of our current results is limited as we did not measure muscle–tendon properties or dynamics, cognitive traits or metabolic cost. We also cannot rule out that the observed adaptations were influenced by trial order, fatigue or other effects that might have dissipated with longer intervals between trials. It would have been informative to test whether adaptations occurred between consecutive BWi trials, whether sequencing BW20 before BW10 produced the same effects in both conditions and in BWf, and whether the same adaptations emerged with more time between trials. Future studies should: (1) prioritise disentangling physical and cognitive sources of interindividual variability in self-selected mechanics; (2) use techniques such as blood flow restriction (Raburn et al., 2011), nerve blocks (Konradsen et al., 1993), muscle or tendon vibration (Hubbuch et al., 2015), or other pharmacological interventions (Amann et al., 2009) that transiently attenuate or intensify specific forms of afferent feedback while measuring self-selected mechanics; and (3) use modelling approaches that begin with the simplest systems capable of simulating locomotion and selectively increase in complexity to identify key components underlying adaptation to novel tasks.

We have demonstrated that vertical hopping and bouncing tasks continue to be a useful experimental framework for research into neuromechanical mechanisms (e.g. Farley et al., 1991; Ferris and Farley, 1997; Farley and Morgenroth, 1999; Moritz and Farley, 2004; Raburn et al., 2011), including sources of individual variation; the role of central and peripheral feedback and feedforward mechanisms; muscle coordination to achieve task-level mechanical demands; and, generally, the effects of perturbing the interactions between external demands, lower-limb kinematics, and muscle–tendon dynamics during spring-like locomotion. Importantly, salient features of the mechanics and energetics of hopping may be effectively modelled using a single muscle–tendon unit, enabling a bridge for experiments across scales – from locomotion to isolated muscle (Robertson and Sawicki, 2015).

Our findings suggest that preferred mechanics of hopping and bouncing can be recalibrated through force exploration. The adaptations observed with added mass exposure may align with a reduction in metabolic cost. Added mass may have allowed our participants to more readily detect the cost gradient through load-sensitive afferent feedback, allowing adaptation toward an energetic optimum (Croft et al., 2019; Dean, 2013; Seethapathi et al., 2024). Alternatively, added mass may have shifted the neuromuscular system's priority landscape toward different criteria such as fatigue avoidance or perceived effort (Bonnard et al., 1994; Huffenus and Forestier, 2006; Singh and Latash, 2011). Our results also suggest that time spent hopping and bouncing – independent of added mass – contributed to the refinement of motor control strategies. Recognising how factors such as added mass and task experience influence locomotor control could contribute towards improving function for individuals with sensorimotor deficits and is essential for refining neuromechanical control models that have applications to predictive musculoskeletal simulations and human–machine interaction.

## Supplementary Material

10.1242/jexbio.250848_sup1 Supplementary information
