# Supplementary material for: Frequency and leg stiffness adaptation in human vertical hopping before, during and after added load
Source: J Exp Biol. 2025 Dec 11;228(24):jeb250848. doi: 10.1242/jeb.250848 (PMC12752494; doi:10.1242/jeb.250848)
Supplement: Supplementary information [file jexbio-228-250848-s1.pdf]

## Supplementary Materials and Methods

On a separate day following the hopping protocol, each participant performed the same initial body weight (BWi), 10% added body mass (BW10), 20% added body mass (BW20), and final body weight (BWf) condition but whilst bouncing in place on both feet (i.e. without an aerial phase). Participants were instructed to oscillate vertically at a comfortable, sustainable rhythm and pace, with bounce frequency and height left unconstrained. We excluded bouncing data from five of the eighteen participants due to an inability to sustain a cyclical bouncing rhythm for the full length of each trial.

Kinematic, ground reaction force, and ankle muscle electromyographic (EMG) data were collected the same way as for hopping. Variables of interest included bounce frequency, leg stiffness, peak vertical force ($F_{peak}$), centre of mass (CoM) work, and mean EMG of lateral gastrocnemius (LG), medial gastrocnemius (MG), soleus (SOL), and tibialis anterior (TA). Due to the lack of aerial phase, the 'bounce cycle' was defined between consecutive positive crossings of ground reaction force (GRF) relative to body weight. Bounce frequency, CoM work, and mean EMG were measured between these crossings. Leg stiffness was again calculated as the ratio of $F_{peak}$ and the average vertical displacement of the pelvis markers from the point where GRF equaled body weight through until $F_{peak}$.

The same statistical approach was performed as with hopping, involving linear mixed-effects ANOVA (Y ~ 1 + condition + sex + age + (condition|ind)) on dimensionless quantities taken from the middle 30-80% portion of each trial. For each variable, we tested for significant effects of condition and sex ($p \leq 0.0209$), assessed pairwise differences between BWi and BW10, BW20, and BWf ($p \leq 0.05$), and computed Cohen's *d* to quantify the effect sizes of these differences.

Journal of Experimental Biology • Supplementary information

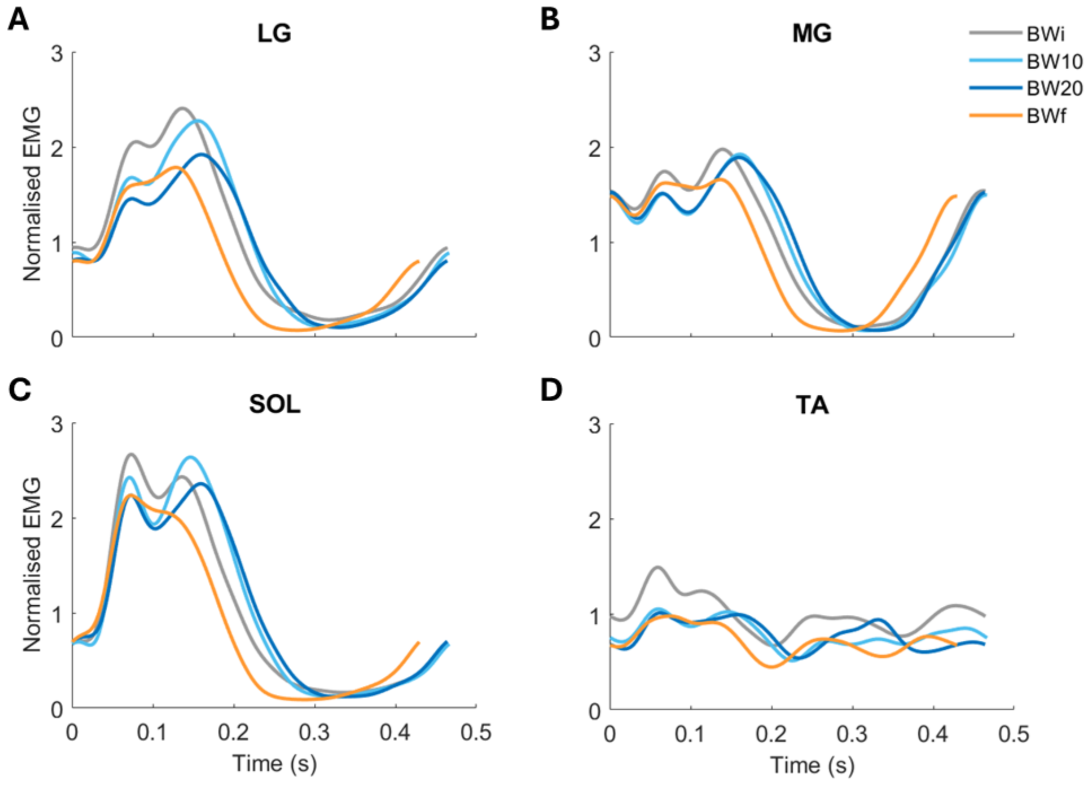


**Fig. S1.** Normalised EMG across the entire hop cycle, averaged across all participants, for (A) lateral gastrocnemius (LG), (B) medial gastrocnemius (MG), (C) soleus (SOL), and (D) tibialis anterior (TA) in response to the body weight (BWi; grey), body weight +10% (BW10; light blue), body weight +20% (BW20; dark blue), and final body weight (BWf; orange) condition.

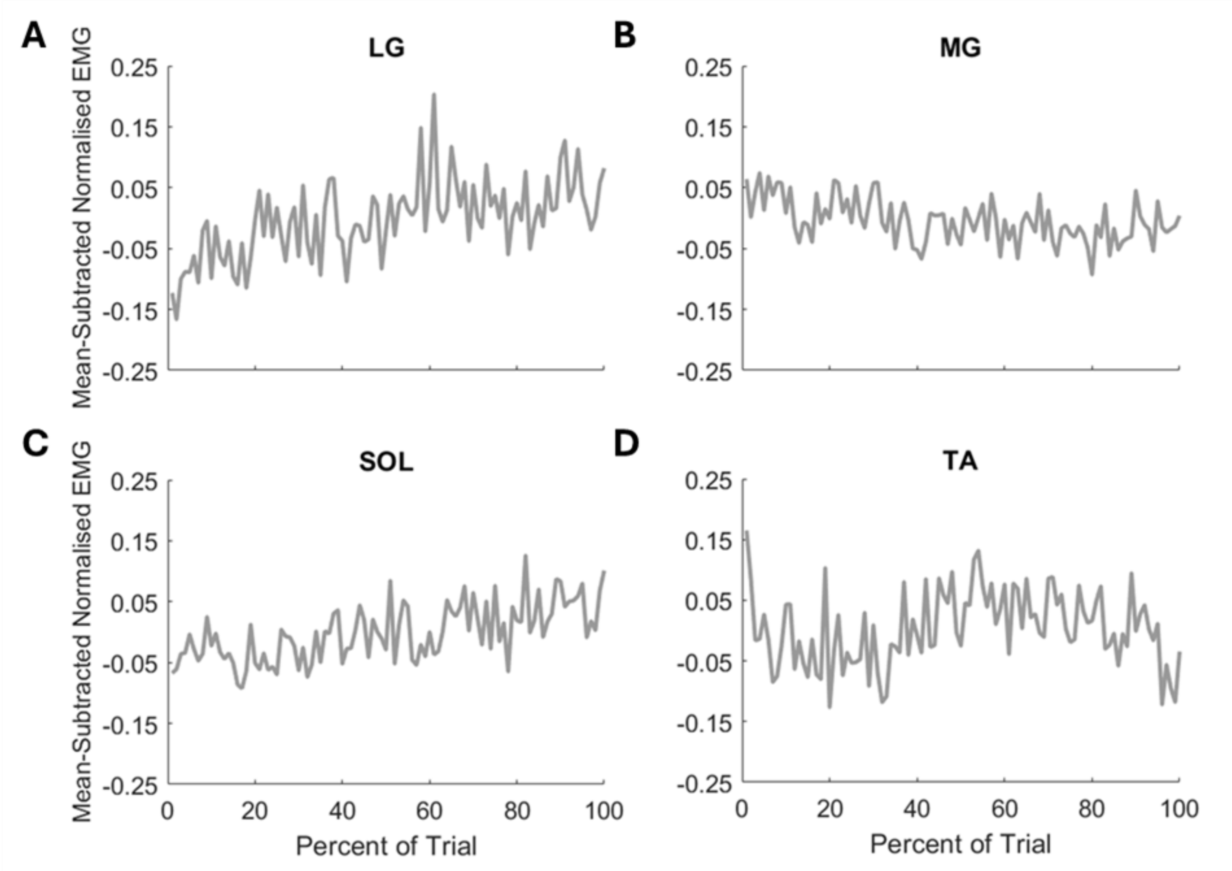


**Fig. S2.** Adaptation of normalised EMG, averaged across all participants, of (**A**) lateral gastrocnemius (LG), (**B**) medial gastrocnemius (MG), (**C**) soleus (SOL), and (**D**) tibialis anterior (TA) over the duration of the initial body weight hopping condition.

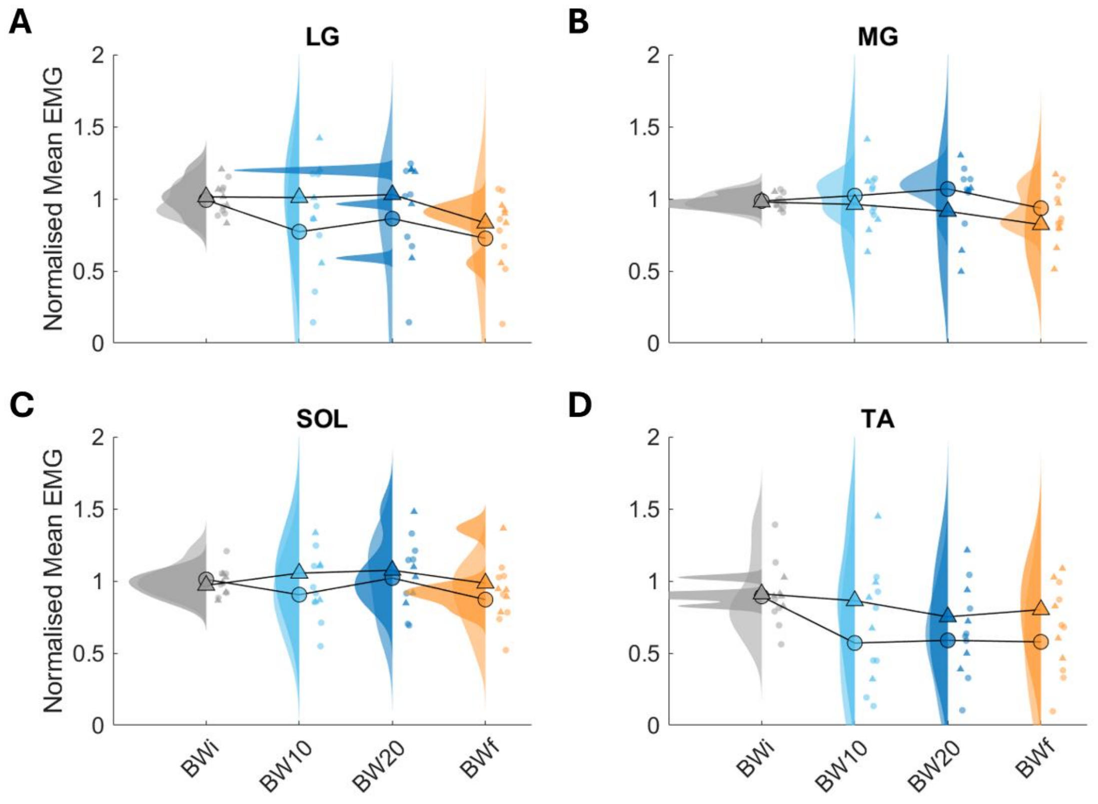


**Fig. S3.** Rain cloud plots of male (circles; lighter rain clouds) and female (triangles; darker rain clouds) mean electromyography (EMG) from (**A**) lateral gastrocnemius (LG), (**B**) medial gastrocnemius (MG), (**C**) soleus (SOL), and (**D**) tibialis anterior (TA) in response the initial body weight (BWi; grey), body weight +10% (BW10; light blue), body weight +20% (BW20; dark blue), and final body weight (BWf; orange) **bouncing** condition. Individual points are the mean for each participant, and the mean values across participants are connected with lines.

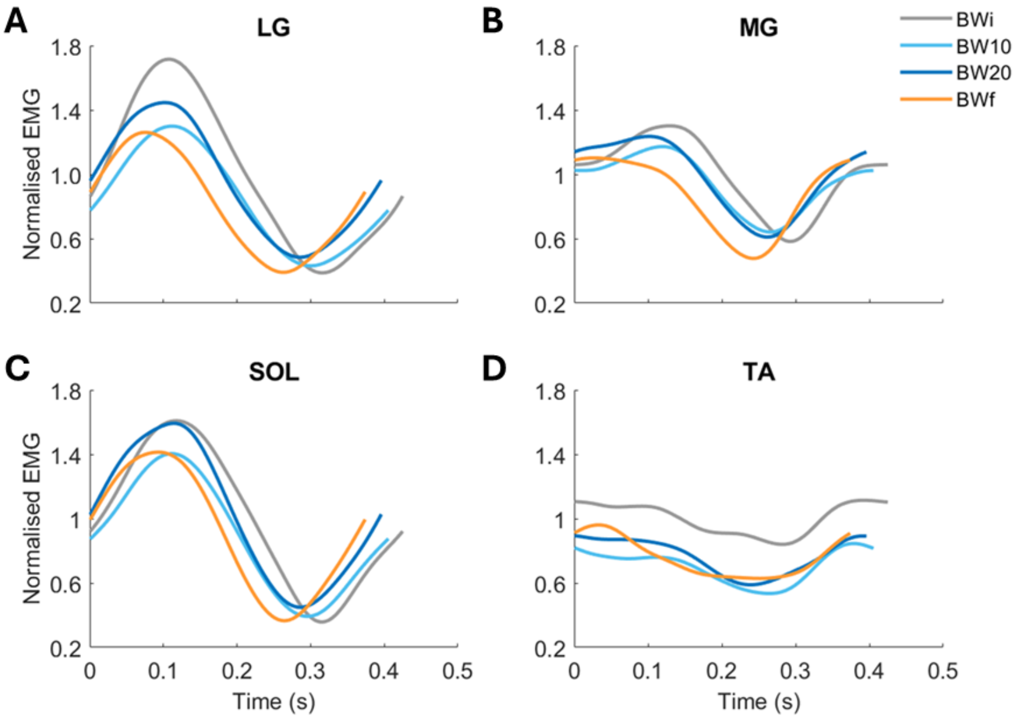


**Fig. S4.** Normalised EMG across the entire **bounce** cycle, averaged across all participants, for (**A**) lateral gastrocnemius (LG), (**B**) medial gastrocnemius (MG), (**C**) soleus (SOL), and (**D**) tibialis anterior (TA) in response to the initial body weight (BWi; grey), body weight +10% (BW10; light blue), body weight +20% (BW20; dark blue), and final body weight (BWf; orange) condition.

**Table S1.** Hopping participant characteristics.

| Participant | Sex | Age | Mass (kg) | Leg Length (m) |
|---|---|---|---|---|
| 1 | F | 21 | 60.3 | 0.87 |
| 2 | F | 21 | 48.2 | 0.82 |
| 3 | M | 26 | 61.4 | 0.94 |
| 4 | F | 44 | 61.7 | 0.83 |
| 5 | F | 35 | 62.5 | 0.90 |
| 6 | F | 28 | 51.8 | 0.79 |
| 7 | M | 29 | 78.9 | 0.90 |
| 8 | M | 31 | 75.5 | 0.89 |
| 10 | F | 62 | 54.9 | 0.80 |
| 11 | M | 19 | 61.7 | 0.87 |
| 12 | M | 35 | 79.4 | 0.89 |
| 13 | M | 42 | 66.5 | 0.97 |
| 14 | M | 28 | 80.8 | 1.01 |
| 15 | M | 21 | 63.2 | 0.93 |
| 16 | F | 26 | 59.9 | 0.85 |
| 17 | M | 47 | 65.7 | 0.86 |
| 18 | M | 21 | 87.6 | 0.90 |

Journal of Experimental Biology • Supplementary information

**Table S2.** Bouncing participant characteristics.

| Participant | Sex | Age | Mass (kg) | Leg Length (m) |
|---|---|---|---|---|
| 1 | F | 21 | 60.3 | 0.87 |
| 3 | M | 26 | 61.4 | 0.94 |
| 4 | F | 44 | 61.7 | 0.83 |
| 5 | F | 35 | 62.5 | 0.90 |
| 6 | F | 28 | 51.8 | 0.79 |
| 8 | M | 31 | 75.5 | 0.89 |
| 10 | F | 62 | 54.9 | 0.80 |
| 11 | M | 19 | 61.7 | 0.87 |
| 12 | M | 35 | 79.4 | 0.89 |
| 13 | M | 42 | 66.5 | 0.97 |
| 14 | M | 28 | 80.8 | 1.01 |
| 15 | M | 21 | 63.2 | 0.93 |
| 17 | M | 47 | 65.7 | 0.86 |

Journal of Experimental Biology • Supplementary information

**Table S3.** Linear mixed-effects ANOVA results and pairwise differences (mean ± 95% CI) from BWi for hopping (dimensionless quantities). Condition, age, and sex were fixed effects, with (condition | individual) as random effects (Model 6, see "Methods"). Degrees of freedom: condition = 3, age = 1, sex = 1, error = 403. Bolding indicates statistical significance.

| ANOVA F-stat | | Variable name | Condition: Pairwise Difference from BWi (mean ± 95% CI (effect size)) | | |
|---|---|---|---|---|---|
| **Condition** | **Sex** | **(Dimensionless)** | **BW10** | **BW20** | **BWf** |
| **14.70** | **7.11** | Hop frequency | -0.0041 ± 0.0066 (-0.09) | 0.0020 ± 0.0066 (0.07) | **0.0582 ± 0.0066 (1.08)** |
| **43.82** | **24.75** | Stance time | **0.0340 ± 0.0073 (0.81)** | **0.0478 ± 0.0073 (0.66)** | **-0.0397 ± 0.0073 (-0.74)** |
| **8.81** | 1.90 | Aerial time | **-0.0237 ± 0.0016 (-0.54)** | **-0.0461 ± 0.0016 (-0.99)** | **-0.0767 ± 0.0016 (-1.05)** |
| **44.72** | **24.63** | Resonant frequency | **-0.0422 ± 0.0076 (-0.93)** | **-0.0530 ± 0.0076 (-0.77)** | **0.0606 ± 0.0076 (0.79)** |
| **18.25** | 5.04 | Duty factor | **0.0189 ± 0.0064 (1.23)** | **0.0295 ± 0.0064 (1.21)** | 0.0051 ± 0.0064 (0.29) |
| **14.39** | 4.75 | Peak vertical force | 0.0513 ± 0.0718 (0.23) | **0.1834 ± 0.0718 (0.61)** | -0.0699 ± 0.0718 (-0.25) |
| **3.20** | **16.53** | Load rate | **-0.3490 ± 0.3129 (-0.35)** | -0.0436 ± 0.3129 (-0.01) | **0.4814 ± 0.3129 (0.25)** |
| **7.32** | 0.55 | Leg stiffness | **0.0018 ± 0.0005 (0.53)** | **0.0060 ± 0.0005 (1.02)** | **0.0051 ± 0.0005 (0.85)** |
| **23.73** | 2.14 | CoM work per hop | 0.0027 ± 0.0036 (0.01) | **0.0065 ± 0.0036 (0.19)** | **-0.0299 ± 0.0036 (-0.95)** |
| **16.22** | 1.04 | CoM avg. power | 0.0012 ± 0.0059 (-0.03) | **0.0068 ± 0.0059 (0.25)** | **-0.0207 ± 0.0059 (-0.79)** |
| 1.26 | 0.02 | CoM peak power | **-0.0485 ± 0.0431 (-0.40)** | **-0.0709 ± 0.0431 (-0.42)** | **-0.0859 ± 0.0431 (-0.51)** |
| **7.45** | 0.38 | Ankle angle at touchdown | **0.0287 ± 0.0155 (0.66)** | **0.0504 ± 0.0155 (1.13)** | **0.0261 ± 0.0155 (0.60)** |
| 1.22 | 0.64 | Knee angle at touchdown | 0.0071 ± 0.0180 (0.27) | 0.0093 ± 0.0180 (0.17) | -0.0024 ± 0.0180 (-0.10) |
| 0.52 | 4.15 | Hip angle at touchdown | 0.0045 ± 0.0219 (0.05) | -0.0041 ± 0.0219 (-0.15) | -0.0130 ± 0.0219 (-0.33) |
| **8.50** | **11.83** | Ankle angular displacement | -0.0167 ± 0.0303 (-0.33) | **-0.0412 ± 0.0303 (-0.57)** | **-0.0750 ± 0.0303 (-1.06)** |
| **7.82** | **5.73** | Knee angular displacement | **0.0316 ± 0.0238 (0.35)** | 0.0214 ± 0.0238 (0.20) | **-0.0433 ± 0.0238 (-0.47)** |

Journal of Experimental Biology • Supplementary information

| | | | | | |
|---|---|---|---|---|---|
| **3.36** | **8.15** | Hip angular displacement | 0.0012 ± 0.0194 (-0.16) | 0.0020 ± 0.0194 (-0.03) | **-0.0310 ± 0.0194 (-0.50)** |
| 2.61 | **8.92** | Ankle stiffness | 0.0113 ± 0.0146 (0.19) | **0.0400 ± 0.0146 (0.49)** | **0.0555 ± 0.0146 (0.52)** |
| 0.24 | 5.22 | Knee stiffness | -0.0040 ± 0.0614 (-0.17) | 0.0160 ± 0.0614 (0.04) | 0.0044 ± 0.0614 (-0.02) |
| 0.19 | 0.03 | Hip stiffness | -0.2558 ± 1.3089 (-0.09) | -0.3658 ± 1.3089 (-0.07) | -0.4213 ± 1.3089 (0.03) |
| 0.92 | 2.47 | Ankle peak moment | 0.0038 ± 0.0098 (0.02) | **0.0106 ± 0.0098 (0.23)** | 0.0029 ± 0.0098 (-0.01) |
| **13.28** | 1.10 | Knee peak moment | 0.0066 ± 0.0069 (0.34) | **0.0123 ± 0.0069 (0.44)** | **-0.0126 ± 0.0069 (-0.53)** |
| **5.66** | 2.38 | Hip peak moment | **-0.0167 ± 0.0118 (-0.70)** | **-0.0135 ± 0.0118 (-0.63)** | **-0.0285 ± 0.0118 (-0.98)** |
| 1.62 | 0.02 | Ankle peak power | **-0.0197 ± 0.0163 (-0.51)** | **-0.0241 ± 0.0163 (-0.38)** | **-0.0252 ± 0.0163 (-0.44)** |
| **4.04** | 1.06 | Knee peak power | -0.0052 ± 0.0084 (-0.26) | -0.0024 ± 0.0084 (-0.12) | **-0.0175 ± 0.0084 (-0.70)** |
| **3.61** | **6.26** | Hip peak power | **-0.0115 ± 0.0055 (-0.74)** | **-0.0098 ± 0.0055 (-0.86)** | **-0.0148 ± 0.0055 (-0.84)** |
| **7.64** | 5.77 | Ankle positive work | -0.0015 ± 0.0034 (-0.23) | -0.0011 ± 0.0034 (-0.14) | **-0.0084 ± 0.0034 (-0.79)** |
| **6.54** | 4.88 | Ankle negative work | -0.0029 ± 0.0034 (-0.26) | **-0.0037 ± 0.0034 (-0.33)** | **0.0049 ± 0.0034 (0.64)** |
| **13.43** | 1.55 | Knee positive work | 0.0003 ± 0.0018 (0.07) | 0.0010 ± 0.0018 (0.19) | **-0.0043 ± 0.0018 (-0.55)** |
| **8.61** | 4.17 | Knee negative work | -0.0015 ± 0.0015 (-0.26) | **-0.0018 ± 0.0015 (-0.33)** | **0.0034 ± 0.0015 (0.64)** |
| **4.16** | **12.03** | Hip positive work | **-0.0012 ± 0.0009 (-0.52)** | -0.0006 ± 0.0009 (-0.29) | **-0.0025 ± 0.0009 (-0.61)** |
| **4.88** | **10.98** | Hip negative work | **0.0012 ± 0.0009 (0.50)** | **0.0013 ± 0.0009 (0.40)** | **0.0026 ± 0.0009 (0.68)** |
| **10.94** | 3.20 | LG mean EMG | -0.0484 ± 0.0536 (-0.20) | **-0.1197 ± 0.0536 (-0.39)** | **-0.2370 ± 0.0536 (-1.02)** |
| **6.77** | 0.16 | MG mean EMG | -0.0359 ± 0.0447 (-0.23) | 0.0112 ± 0.0447 (-0.17) | **-0.0683 ± 0.0447 (-0.47)** |
| **11.68** | 2.72 | SOL mean EMG | 0.0354 ± 0.0503 (0.39) | 0.0440 ± 0.0503 (0.33) | **-0.1298 ± 0.0503 (-0.89)** |
| **13.94** | 1.99 | TA mean EMG | **-0.2494 ± 0.0724 (-1.16)** | **-0.2187 ± 0.0724 (-0.90)** | **-0.3000 ± 0.0724 (-1.44)** |

Journal of Experimental Biology • Supplementary information

**Table S4.** Pairwise differences (mean ± 95% CI) from BWi for hopping (SI quantities). F-stat values are the same as in Table S3. Condition, age, and sex were fixed effects, with (condition | individual) as random effects (Model 6, see "Methods"). Degrees of freedom: condition = 3, age = 1, sex = 1, error = 403. Bolding indicates statistical significance.

| **Variable name** | **Condition: Pairwise Difference from BWi (mean ± 95% CI)** | | |
|---|---|---|---|
| **(SI units)** | **BW10** | **BW20** | **BWf** |
| Hop frequency (Hz) | -0.01 ± 0.02 | 0.01 ± 0.02 | **0.19 ± 0.02** |
| Stance time (s) | **0.01 ± 0.002** | **0.01 ± 0.002** | **-0.01 ± 0.002** |
| Air time (s) | **-0.01 ± 0.001** | **-0.01 ± 0.001** | **-0.02 ± 0.001** |
| Resonant frequency (Hz) | **-0.14 ± 0.02** | **-0.18 ± 0.02** | **0.20 ± 0.02** |
| Duty factor | **0.0189 ± 0.0064** | **0.0295 ± 0.0064** | 0.0051 ± 0.0064 |
| Peak vertical force (N) | 33.11 ± 46.34 | **118.36 ± 46.34** | -45.11 ± 46.34 |
| Load rate (N·s$^{-1}$) | **-749.77 ± 672.21** | -93.67 ± 672.21 | **1034.21 ± 672.21** |
| Leg stiffness (kN·m$^{-1}$) | **1.03 ± 0.29** | **3.42 ± 0.29** | **2.91 ± 0.29** |
| CoM work per hop (J) | 1.54 ± 2.05 | **3.71 ± 2.05** | **-17.06 ± 2.05** |
| CoM avg. power (W) | 2.28 ± 11.20 | **12.91 ± 11.20** | **-39.31 ± 11.20** |
| CoM peak power (W) | **-92.11 ± 81.85** | **-134.65 ± 81.85** | **-163.13 ± 81.85** |
| Ankle angle at touchdown (deg) | **1.64 ± 0.89** | **2.89 ± 0.89** | **1.5 ± 0.89** |
| Knee angle at touchdown (deg) | 0.41 ± 1.03 | 0.53 ± 1.03 | -0.14 ± 1.03 |
| Hip angle at touchdown (deg) | 0.26 ± 1.25 | -0.23 ± 1.25 | -0.74 ± 1.25 |
| Ankle angular displacement (deg) | -0.96 ± 1.74 | **-2.36 ± 1.74** | **-4.3 ± 1.74** |
| Knee angular displacement (deg) | **1.81 ± 1.36** | 1.23 ± 1.36 | **-2.48 ± 1.36** |
| Hip angular displacement (deg) | 0.07 ± 1.11 | 0.11 ± 1.11 | **-1.78 ± 1.11** |
| Ankle stiffness (Nm·rad$^{-1}$) | 6.45 ± 8.33 | **22.82 ± 8.33** | **31.66 ± 8.33** |

| | | | |
|---|---|---|---|
| Knee stiffness (Nm·rad$^{-1}$) | -2.28 ± 35.03 | 9.13 ± 35.03 | 2.51 ± 35.03 |
| Hip stiffness (Nm·rad$^{-1}$) | -145.93 ± 746.73 | -208.69 ± 746.73 | -240.35 ± 746.73 |
| Ankle peak moment (Nm) | 2.17 ± 5.59 | **6.05 ± 5.59** | 1.65 ± 5.59 |
| Knee peak moment (Nm) | 3.77 ± 3.94 | **7.02 ± 3.94** | **-7.19 ± 3.94** |
| Hip peak moment (Nm) | **-9.53 ± 6.73** | **-7.70 ± 6.73** | **-16.26 ± 6.73** |
| Ankle peak power (W) | **-37.41 ± 30.96** | **-45.77 ± 30.96** | **-47.86 ± 30.96** |
| Knee peak power (W) | -9.88 ± 15.95 | -4.56 ± 15.95 | **-33.23 ± 15.95** |
| Hip peak power (W) | **-21.84 ± 10.45** | **-18.61 ± 10.45** | **-28.11 ± 10.45** |
| Ankle positive work (J) | -0.86 ± 1.94 | -0.63 ± 1.94 | **-4.79 ± 1.94** |
| Ankle negative work (J) | -1.65 ± 1.94 | **-2.11 ± 1.94** | **2.8 ± 1.94** |
| Knee positive work (J) | 0.17 ± 1.03 | 0.57 ± 1.03 | **-2.45 ± 1.03** |
| Knee negative work (J) | -0.86 ± 0.86 | **-1.03 ± 0.86** | **1.94 ± 0.86** |
| Hip positive work (J) | **-0.68 ± 0.51** | -0.34 ± 0.51 | **-1.43 ± 0.51** |
| Hip negative work (J) | **0.68 ± 0.51** | **0.74 ± 0.51** | **1.48 ± 0.51** |

Journal of Experimental Biology • Supplementary information

**Table S5.** Linear mixed-effects ANOVA results and pairwise differences (mean ± 95% CI) from BWi for **bouncing** (dimensionless quantities). Condition, age, and sex were fixed effects, with (condition | individual) as random effects (Model 6, see "Methods"). Degrees of freedom: condition = 3, age = 1, sex = 1, error = 313. Bolding indicates statistical significance.

| ANOVA F-stat | | | Variable name | Condition: Pairwise Difference from BWi (mean ± 95% CI (effect size)) | | |
|---|---|---|---|---|---|---|
| **Condition** | **Age** | **Sex** | **(Dimensionless)** | **BW10** | **BW20** | **BWf** |
| **29.11** | **12.95** | **6.34** | Bounce frequency | 0.0196 ± 0.0295 (0.28) | **0.0371 ± 0.0295 (0.48)** | **0.0893 ± 0.0295 (1.60)** |
| **61.00** | **6.06** | **5.17** | Peak vertical force | **0.1513 ± 0.0385 (1.53)** | **0.3084 ± 0.0385 (1.99)** | **0.0954 ± 0.0385 (0.61)** |
| **12.73** | 0.02 | 2.42 | Leg stiffness | **0.0111 ± 0.0024 (1.06)** | **0.0233 ± 0.0024 (1.38)** | **0.0045 ± 0.0024 (0.32)** |
| 2.49 | 0.06 | 3.67 | CoM work per bounce | 0.0011 ± 0.0019 (0.16) | **0.0056 ± 0.0019 (0.46)** | 0.0001 ± 0.0019 (0.04) |
| **5.67** | 0.57 | 0.001 | LG mean EMG | -0.0987 ± 0.1128 (-0.31) | -0.0568 ± 0.1128 (-0.18) | **-0.2005 ± 0.1128 (-0.73)** |
| **8.63** | 0.0007 | 0.92 | MG mean EMG | 0.0213 ± 0.0665 (0.06) | 0.0148 ± 0.0665 (0.09) | **-0.0970 ± 0.0665 (-0.43)** |
| **6.31** | 2.24 | 0.48 | SOL mean EMG | -0.0463 ± 0.0718 (-0.11) | 0.0467 ± 0.0718 (0.20) | -0.0709 ± 0.0718 (-0.35) |
| 0.90 | 0.19 | 0.01 | TA mean EMG | **-0.2292 ± 0.1996 (-0.41)** | **-0.2164 ± 0.1996 (-0.56)** | -0.1881 ± 0.1996 (-0.51) |

**Table S6.** Pairwise differences (mean ± 95% CI) from BWi for **bouncing** (SI quantities). F-stat values are the same as in Table S5. Condition, age, and sex were fixed effects, with (condition | individual) as random effects (Model 6, see "Methods"). Degrees of freedom: condition = 3, age = 1, sex = 1, error = 313. Bolding indicates statistical significance.

| Variable name | Condition: Pairwise Difference from BWi (mean ± 95% CI) | | |
|---|---|---|---|
| **(SI units)** | **BW10** | **BW20** | **BWf** |
| Bounce frequency (Hz) | 0.06 ± 0.10 | **0.12 ± 0.10** | **0.30 ± 0.10** |
| Peak vertical force (N) | **96.38 ± 24.53** | **196.46 ± 24.53** | **60.77 ± 24.53** |
| Leg stiffness (kN·m$^{-1}$) | **6.28 ± 1.36** | **13.19 ± 1.36** | **2.55 ± 1.36** |
| CoM work per bounce (J) | 0.62 ± 1.08 | **3.17 ± 1.08** | 0.06 ± 1.08 |

Journal of Experimental Biology • Supplementary information
